# Fully Digital vs. Conventional Planning in Bimaxillary Orthognathic Surgery: Effects on 3D Accuracy and Surgical Efficiency

**DOI:** 10.3390/diagnostics16091365

**Published:** 2026-04-30

**Authors:** Petrică Florin Sava, Bogdan Radu Dragomir, Ilie Cristian Drochioi, Otilia Boișteanu, Andrei Nicolau, Daniela Șulea, Ștefan Gherasimescu, Victor Vlad Costan

**Affiliations:** Grigore T. Popa University of Medicine and Pharmacy, 16 Universitatii Str., 700115 Iasi, Romania; petrica-florin.sava@umfiasi.ro (P.F.S.); otilia.boisteanu@umfiasi.ro (O.B.); nicolau.andrei@umfiasi.ro (A.N.); daniela.d.sulea@umfiasi.ro (D.Ș.); stefan-gherasimescu@umfiasi.ro (Ș.G.); victor.costan@umfiasi.ro (V.V.C.)

**Keywords:** bimaxillary orthognathic surgery, CBCT, digital surgical planning, Le Fort I osteotomy, BSSO, three-dimensional accuracy, operative time

## Abstract

**Background**: Accurate transfer of the surgical plan is essential in bimaxillary orthognathic surgery, where small discrepancies between planned and postoperative skeletal positions may influence functional and aesthetic outcomes. This study compared the three-dimensional (3D) accuracy and time efficiency of conventional and fully digital planning workflows using CBCT-based evaluation. **Methods**: This retrospective comparative study included 100 adult patients with skeletal Class II or III malocclusion treated by Le Fort I osteotomy combined with bilateral sagittal split osteotomy (BSSO). Patients were allocated to conventional planning (*n* = 50) or fully digital planning using IPS CaseDesigner (*n* = 50). Planned and postoperative CBCT datasets were superimposed using voxel-based cranial base registration. Linear deviations at A-point and Pogonion, total RMS error, planning time, and operative time were analyzed. **Results**: Mean deviations were lower in the digital group at A-point (1.28 ± 0.28 mm vs. 1.63 ± 0.36 mm) and Pogonion (1.49 ± 0.42 mm vs. 1.95 ± 0.44 mm) (*p* < 0.001). Total RMS deviation was reduced in the digital workflow (1.39 ± 0.39 mm vs. 1.80 ± 0.54 mm; *p* < 0.001). Planning and operative times were significantly shorter in the digital group (*p* < 0.001). Moderate correlations were observed between time variables and 3D deviations. **Conclusions**: Fully digital planning showed improved 3D accuracy and significantly reduced planning and operative times compared with conventional methods, while maintaining clinically acceptable deviations.

## 1. Introduction

Bimaxillary orthognathic surgery represents a complex intervention in which the accuracy of preoperative planning constitutes one of the essential conditions for achieving a stable functional outcome and adequate facial harmony. The correct three-dimensional positioning of the maxillary and mandibular segments directly influences occlusal balance, the functional relationships of the temporomandibular joint, and facial aesthetics. For this reason, precise surgical planning represents a critical step in the management of dentofacial deformities and in ensuring postoperative stability [1,2].

Conventional orthognathic planning methods are based on the fabrication of plaster study models of the dental arches, mounted on a mechanical articulator and manually manipulated to simulate the displacement of bony segments. This approach provides direct control over occlusal contacts and allows the evaluation of intermaxillary relationships in a manner familiar to the clinician. However, the process involves several error-prone steps, including deformation of impression materials, inaccurate transfer of occlusal relationships into the articulator, and manual adaptation of surgical splints. In addition, the reproducibility of the outcome largely depends on the operator’s experience and the accuracy of laboratory procedures [3,4].

Technological advances over the past two decades have enabled the integration of three-dimensional imaging into orthognathic surgical planning. Cone-beam computed tomography (CBCT) provides detailed information on craniofacial anatomy, allowing volumetric evaluation of bone structures and three-dimensional analysis of dentofacial deformities [5]. The fusion of CBCT images with dental models obtained through intraoral scanning or digitized plaster casts allows the creation of a complete virtual patient model, which can be used for simulating osteotomies and planning the repositioning of bony segments in relation to the cranial base [6].

Dedicated virtual surgical planning software, such as IPS CaseDesigner (KLS Martin, Tuttlingen, Germany) or NemoFAB (Nemotec, Leganés, Spain), enables three-dimensional analysis of cephalometric parameters, simulation of osteotomies, and evaluation of dentoskeletal relationships in a reproducible manner. Furthermore, the integration of 3D printing technology allows the fabrication of surgical guides and customized occlusal splints, reducing manual intervention and increasing the concordance between planning and the final outcome [7,8,9]. Recent studies have shown that fully digital workflows can improve the predictability of maxillary and mandibular positioning and reduce intraoperative variability [10,11,12].

The literature reports variations in the accuracy of these methods, particularly in bimaxillary surgery, where cumulative errors may influence the final outcome. Strujak et al. reported a mean deviation of approximately 1.5 mm between virtual planning and postoperative results in fully digital workflows, compared with approximately 1.9 mm for conventional methods [13]. Nilsson et al. confirmed that differences between methods remain below the clinically acceptable threshold of 2 mm; however, result consistency is higher when the entire workflow is digitalized [14]. Similarly, Zhou et al. demonstrated improved predictability of maxillary positioning in digital planning, although absolute differences compared to conventional methods are not always statistically significant [15]. Other studies have shown that integrating intraoral scans with CBCT data contributes to increased accuracy of three-dimensional model alignment and reduced positioning errors [16,17].

In addition to geometric accuracy, time efficiency represents an important parameter in the evaluation of surgical workflows. Reducing the number of laboratory steps and enabling rapid modifications in a virtual environment can contribute to shortening planning time and optimizing intraoperative stages. Recent studies have shown that integrating digital workflows can reduce operative time by approximately 20–30% by decreasing the need for intraoperative adjustments and improving the precision of surgical guides [18,19,20]. This reduction in operative time may contribute to improved intraoperative management and increased predictability of the surgical outcome.

The aim of the present study was to compare the three-dimensional accuracy and time efficiency of conventional and fully digital surgical planning in bimaxillary orthognathic surgery (Le Fort I osteotomy combined with bilateral sagittal split osteotomy—BSSO). The analysis focused on evaluating three-dimensional deviations at the level of the cephalometric landmarks A-point and Pogonion, as well as comparing planning and operative times.

The null hypothesis (H_0_) assumes that there are no statistically significant differences between conventional and fully digital planning in terms of either three-dimensional accuracy or operative duration.

The alternative hypothesis (H_1_) assumes that fully digital planning results in smaller three-dimensional deviations between planning and outcome and leads to a reduction in operative time compared to the conventional method.

While previous studies have reported improvements in either accuracy or time efficiency with digital workflows, these outcomes are often evaluated separately and in heterogeneous samples. The present study provides a comparative assessment of both parameters within a homogeneous cohort of bimaxillary orthognathic surgery cases treated in a single center.

## 2. Materials and Methods

### 2.1. Study Design

**Figure 1:**
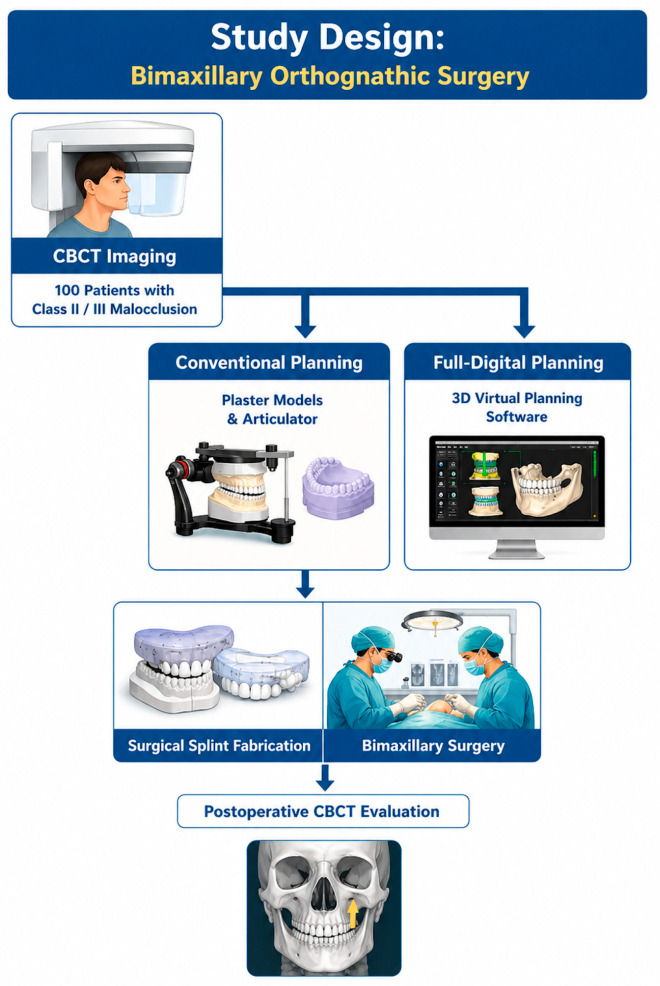
Study design.

The study was conducted as a retrospective comparative study, aiming to evaluate differences in three-dimensional accuracy and time efficiency between conventional surgical planning and fully digital planning in bimaxillary orthognathic surgery. This design allowed the analysis of outcomes from previously performed surgical interventions by comparing the concordance between preoperative planning and the postoperative position of bony segments. The retrospective approach was appropriate for investigating the relationship between the planning method used and the accuracy of surgical plan transfer, as well as for evaluating its impact on planning and operative times. The design of the study is presented in Figure 1.

### 2.2. Participants and Selection Criteria

The study included patients diagnosed with skeletal Class II or Class III dentofacial deformities, treated by bimaxillary orthognathic surgery (Le Fort I osteotomy combined with bilateral sagittal split osteotomy of the mandibular ramus—BSSO). Cases were retrospectively selected from the database of an oral and maxillofacial surgery center.

The study was conducted in accordance with the principles of the Declaration of Helsinki and was approved by the Ethics Committee of the “Sf. Spiridon” County Emergency Clinical Hospital, Iași, Romania (protocol no. 130/10.09.2025). Written informed consent was obtained from all patients prior to inclusion in the study.

A statistical power analysis was performed to estimate sample size for the comparison of two independent means, using total three-dimensional deviation (RMS) as the primary variable. The calculation was performed for a significance level of 5% and a statistical power of 80%, considering a minimum clinically relevant difference of approximately 0.4 mm between the two planning methods. Based on these parameters, approximately 45–50 patients per group were required. Ultimately, 101 consecutive patients treated between January 2025 and August 2025 were evaluated; one case was excluded due to incomplete data, resulting in a final sample of 100 patients. This was a retrospective study. Data collection and analysis were performed only after ethics approval was obtained.

All patients underwent complete presurgical orthodontic treatment, including full dental decompensation, according to the standard therapeutic protocol for orthognathic surgery. Depending on the planning method used, patients were allocated into two groups:

Conventional planning group (*n* = 50), in which surgical planning was performed on plaster dental models mounted in a mechanical articulator.

Fully digital planning group (*n* = 50), in which planning was performed virtually using CBCT data and digital dental models, with dedicated three-dimensional planning software (IPS CaseDesigner^®^, KLS Martin, Tuttlingen, Germany).

Patient allocation into the two groups was determined by the workflow available at the time of surgical planning. The transition from conventional to fully digital planning occurred as part of a center-level workflow change, resulting in a chronological separation between the two groups. All surgeries were performed by the same surgical and orthodontic team, using a standardized clinical protocol to reduce operator variability.

*Inclusion Criteria*

Patients meeting the following criteria were included:

- Age ≥ 18 years,
- Diagnosis of skeletal Class II or Class III malocclusion,
- Treatment by bimaxillary orthognathic surgery (Le Fort I + BSSO),
- Completed presurgical orthodontic treatment,
- Availability of both preoperative CBCT and postoperative CBCT performed 6–8 weeks after surgery,
- Complete surgical planning documentation.

*Exclusion Criteria*

The following were excluded:

- Craniofacial syndromes and congenital malformations,
- Severe post-traumatic deformities,
- Previous orthognathic surgery,
- CBCT images with artifacts or insufficient quality,
- Incomplete documentation for any of the analyzed stages,
- Additional surgical procedures that could influence maxillomandibular positioning,
- Extensive untreated edentulism,
- Active temporomandibular joint pathology.

Case selection allowed the formation of a relatively homogeneous sample, suitable for comparing three-dimensional accuracy and time efficiency between the two surgical planning methods.

Preoperative CBCT was performed after completion of presurgical orthodontic treatment and achievement of dental decompensation.

### 2.3. Conventional Planning Protocol

For cases included in the conventional planning group, dental impressions were obtained using addition silicone material and cast in type IV hard plaster to produce study models. The maxillary and mandibular models were mounted in a semi-adjustable articulator using a facebow, allowing reproduction of occlusal relationships and maxillary position relative to the cranial base.

Simulation of osteotomies and repositioning of bony segments were performed manually on the study models, aiming to achieve stable occlusal relationships and an appropriate aesthetic outcome. The final intermaxillary position was stabilized using modeling wax, and surgical splints were fabricated from acrylic resin through thermoforming using a controlled pressure system.

This method involves sequential laboratory steps and manual transfer of the surgical plan during the intraoperative stage.

### 2.4. Digital Planning Protocol

In the fully digital planning group, the workflow integrated data obtained from CBCT examination and intraoral scanning. CBCT images were acquired with a voxel resolution of 0.3 mm, while dental surfaces were recorded using intraoral scanning. The datasets were merged to generate a complete three-dimensional virtual model of the craniofacial complex.

Simulation of osteotomies (Le Fort I and BSSO) and repositioning of bony segments were performed virtually using dedicated three-dimensional surgical planning software IPS CaseDesigner^®^, version 2.3.5.2 (KLS Martin, Tuttlingen, Germany). The software allows real-time evaluation of cephalometric relationships and adjustment of segment positioning relative to the cranial base.

Based on the virtual planning, surgical splints and positioning guides were generated and fabricated with 3D printing using biocompatible resin. This workflow enables accurate transfer of the virtual plan to the surgical stage and reduces the need for intraoperative adjustments.

### 2.5. Imaging Protocol and Three-Dimensional Evaluation

CBCT examinations were performed both preoperatively and postoperatively using the same imaging protocol (voxel size: 0.3 mm).

CBCT scans were acquired using ORTHOPANTOMOGRAPH OP 3D PRO cone-beam computed tomography (Kavo Dental, Biberach, Biberach, Germany), with a field of view of 6 cm, voxel size of 0.3 mm, and exposure time of 40 s. All scans were performed using the same protocol to ensure consistency between preoperative and postoperative datasets.

Postoperative imaging was conducted 6–8 weeks after surgery, a time interval selected to minimize the influence of postoperative edema and to allow initial stabilization of the bony segments. This interval was chosen to evaluate early transfer accuracy between the surgical plan and postoperative skeletal position, rather than long-term stability.

Imaging data were exported in DICOM format and analyzed using IPS CaseDesigner software. The three-dimensional models corresponding to the surgical plan and postoperative outcome were superimposed using a voxel-based registration method at the level of the anterior cranial base, a region considered anatomically stable and minimally affected by surgery. This method is commonly used in orthognathic accuracy studies, as it enables stable alignment of three-dimensional models and reduces variability associated with manual identification of anatomical landmarks.

This superimposition method reduces operator-dependent variability compared to approaches based exclusively on anatomical landmarks.

**Figure 2:**
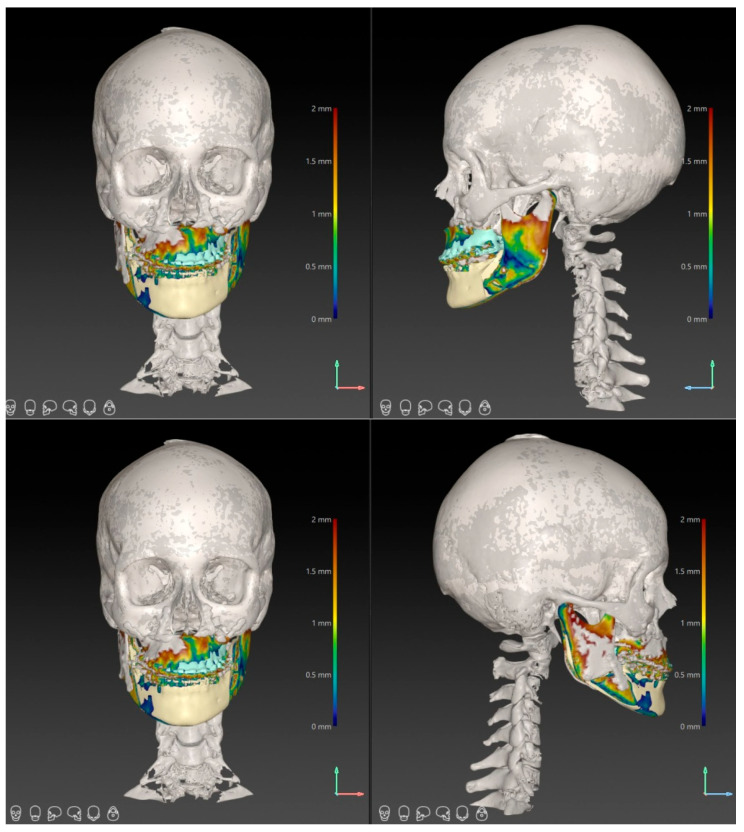
Three-dimensional superimposition between planned and postoperative CBCT models using voxel-based cranial base registration. The superimposition illustrates the spatial concordance between the surgical plan and postoperative skeletal positions at the maxillary (A-point) and mandibular (Pogonion) landmarks.

Three-dimensional superimposition between planned and postoperative CBCT datasets was performed using voxel-based cranial base registration, as illustrated in Figure 2.

### 2.6. Evaluated Parameters

The accuracy of surgical plan transfer was assessed by measuring three-dimensional linear deviations at A-point and Pogonion, representing clinically relevant landmarks for maxillary and mandibular positioning. Additionally, the root mean square (RMS) value was calculated, providing a global measure of three-dimensional discrepancy between planned and postoperative models.

The RMS value was calculated as the root mean square of point-to-point distances between the planned and postoperative surfaces after voxel-based registration, representing the overall three-dimensional discrepancy between models.

Variables related to time efficiency were also analyzed:

Planning time (minutes)—the time required to complete the surgical plan and fabricate surgical splints.

Operative time (minutes)—the interval between the initial incision and completion of suturing.

Operative time did not include anesthesia induction or postoperative recovery.

### 2.7. Measurement Reproducibility

Three-dimensional measurements were performed independently by two evaluators, with each set of measurements repeated twice to assess consistency. Inter- and intra-observer reproducibility were evaluated using the intraclass correlation coefficient (ICC), with obtained values indicating excellent agreement (ICC > 0.90).

Intraclass correlation coefficients ranged from 0.91 to 0.96, indicating excellent agreement. A two-way mixed-effects model with absolute agreement was used.

The measurement tolerance of the software used was ±0.1 mm.

### 2.8. Statistical Analysis

Three-dimensional deviations were calculated using IPS CaseDesigner software by automatically superimposing postoperative CBCT models onto the corresponding planned models, using the cranial base as a reference. For each case, three-dimensional linear deviations (mm) were determined at the level of A-point and Pogonion, along with the global RMS value, representing the total discrepancy between planned and postoperative positions of the bony segments.

Statistical analysis was performed using SPSS Statistics v.29.0 (IBM Corp., Armonk, NY, USA). The distribution of continuous variables was assessed using the Shapiro–Wilk test. Independent samples *t*-tests were used to compare mean values between the conventional and digital planning groups.

The relationship between time efficiency and three-dimensional accuracy was evaluated using bivariate correlation analysis. Pearson’s correlation coefficient (r) was used for normally distributed variables to assess the association between total RMS deviation and planning and operative times.

To identify factors associated with total three-dimensional deviation, a multiple linear regression analysis was performed including planning method (conventional vs. digital), patient age, and skeletal class as independent variables. Planning time was not included in the final model because of its strong association with planning method, which could reduce model interpretability.

Results were expressed as mean ± standard deviation for continuous variables and as frequencies for categorical variables. Statistical significance was set at *p* < 0.05 for all tests.

## 3. Results

### 3.1. Sample Characteristics

A total of 100 patients were included in the study, with ages ranging from 18 to 36 years (mean: 25.02 ± 5.03 years). These values indicate a predominance of young adult patients, a population in which orthognathic surgery is commonly indicated after completion of craniofacial growth.

The sex distribution was relatively balanced, with 54% female and 46% male patients. This distribution does not suggest significant sex-related differences that could influence the study outcomes.

From a skeletal diagnosis perspective, 58% of patients presented with Class II malocclusion, while 42% had Class III. The absence of Class I cases is justified by the specific indication of orthognathic surgery for the correction of significant sagittal discrepancies. The observed distribution is consistent with the typical case profile treated in bimaxillary orthognathic surgery.

Patients were equally distributed between the two study groups, with 50 cases in the conventional planning group and 50 in the fully digital planning group. This balanced allocation allows direct comparison between the two methods under similar conditions, reducing the potential for bias related to unequal group sizes.

Overall, the baseline characteristics of the sample indicate a relatively homogeneous cohort, suitable for comparative analysis of three-dimensional accuracy and time efficiency between the two surgical planning approaches (Table 1).

### 3.2. Baseline Comparability Between Groups

Comparison of age between patients in the conventional planning group and those in the digital planning group showed no statistically significant differences (*p* = 0.502) (Table 2). The mean age was 24.68 ± 5.66 years in the conventional group and 25.36 ± 4.36 years in the digital group, with similar values suggesting a comparable distribution of this variable between the two cohorts.

Sex distribution was also comparable, with no statistically significant differences between groups (*p* = 0.688). The conventional planning group included 28 female and 22 male patients, while the digital group included 26 female and 24 male patients, with similar proportions observed.

Regarding skeletal diagnosis, the distribution of Class II and Class III cases was similar between the two groups (*p* = 0.685). In the conventional group, 28 patients were classified as Class II and 22 as Class III, whereas in the digital group, 30 patients were Class II and 20 were Class III.

Overall, the analyzed variables showed a similar distribution across the two groups, with no statistically significant differences. This indicates that the groups can be considered comparable at baseline. Under these conditions, any subsequent differences identified can be interpreted in relation to the planning method used, without the influence of major variations in baseline patient characteristics (Table 2).

### 3.3. Three-Dimensional Accuracy Analysis

The analysis of three-dimensional accuracy revealed differences between the two surgical planning methods (Table 3). Total RMS values were lower in the digital planning group (1.39 ± 0.39 mm) compared with the conventional planning group (1.80 ± 0.54 mm), with the difference being statistically significant (*p* < 0.001) (Table 3).

A similar trend was observed for the analyzed anatomical landmarks. The mean deviation at A-point was 1.28 ± 0.28 mm in the digital group, compared with 1.63 ± 0.36 mm in the conventional group (*p* < 0.001). Regarding Pogonion, the mean deviation was 1.49 ± 0.42 mm for digital planning and 1.95 ± 0.44 mm for conventional planning, with the difference also being statistically significant (*p* < 0.001).

**Figure 3:**
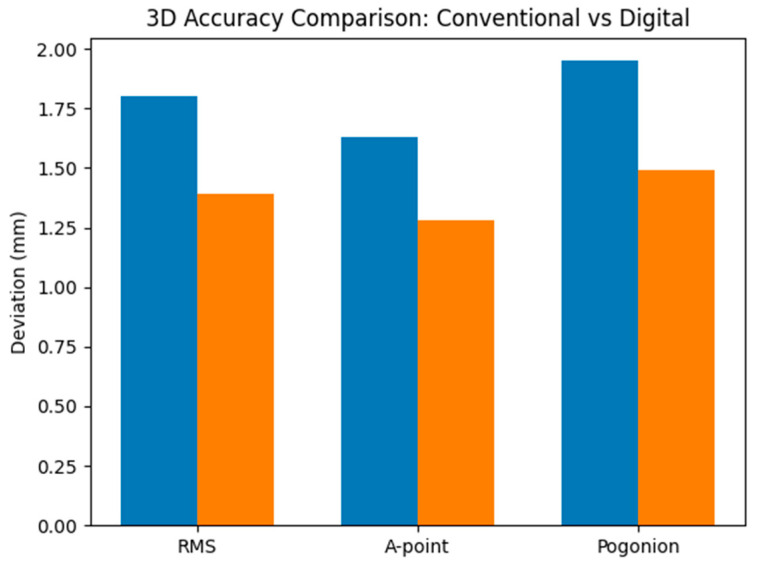
Bar-chart representation of mean three-dimensional deviations according to planning method. Blue—Conventional; Orange—Digital.

A heatmap representation of mean three-dimensional deviations highlights the consistently lower values observed in the fully digital planning group across all evaluated parameters (Figure 3).

The lower deviation values observed in the digital group indicate better concordance between the virtual plan and the postoperative outcome, suggesting higher predictability in the positioning of bony segments. Although the observed differences remain within clinically acceptable limits, they demonstrate reduced variability in the fully digital workflow.

Overall, the results support the idea that digital planning provides improved control of three-dimensional positioning in bimaxillary orthognathic surgery, reducing discrepancies between the planned and final outcomes.

### 3.4. Time Efficiency Analysis

The analysis of planning and operative durations revealed significant differences between the two methods. The mean planning time was significantly longer in the conventional group (163.92 ± 17.05 min) compared with the digital workflow (106.36 ± 13.89 min), with the difference being statistically significant (*p* < 0.001) (Table 4).

A similar difference was observed for operative time. In the conventional planning group, the mean operative time was 277.40 ± 28.29 min, whereas in the digital group it was 184.24 ± 21.16 min, with the difference also reaching statistical significance (*p* < 0.001).

These findings indicate a substantial reduction in both planning and operative time when a fully digital workflow is used. The observed differences suggest improved workflow efficiency and enhanced procedural predictability, with a reduced need for intraoperative adjustments.

The differences identified between the two methods were not only statistically significant but also of considerable magnitude. For total RMS deviation, a large effect size was observed (Cohen’s d ≈ 0.86), while for deviations at A-point and Pogonion, values exceeded the threshold associated with a large effect (d > 1). Regarding workflow duration, the differences between the two planning approaches were even more pronounced, with a very large effect size (d > 3), reflecting a clear reduction in both planning and operative time when using the fully digital workflow.

### 3.5. Multivariable Analysis of Factors Associated with 3D Accuracy

To evaluate factors associated with total three-dimensional deviation, a multiple linear regression model was constructed including planning method, age, and skeletal class (Table 5).

The model was statistically significant (F(3,96) = 7.898, *p* < 0.001) and explained approximately 25% of the variance in RMS values (R^2^ = 0.250).

The planning method showed the strongest association, with lower deviation values in the digital group (*p* < 0.001). Planning time was also statistically associated with RMS values (*p* = 0.010); however, this finding should be interpreted cautiously, given the relationship between planning time and workflow type. Age had a modest effect (*p* = 0.039), while skeletal class was not significantly associated with deviation (*p* = 0.435).

### 3.6. Robustness of the Statistical Model

To assess the stability of the regression model, residuals and confidence intervals of the coefficients were examined. The confidence intervals corresponding to the group and planning time variables did not include zero, supporting the presence of a consistent relationship between these variables and total three-dimensional deviation. The standard error of the estimate (0.45) indicates moderate variability of RMS values around the model, with no evidence suggesting instability of the estimates or the influence of extreme values.

Overall, the obtained results indicate that the use of a fully digital workflow is associated with reduced three-dimensional deviations and a clear shortening of both planning and operative stages. The observed relationships between variables suggest that the reduction in working time is not achieved at the expense of accuracy; on the contrary, it is accompanied by improved concordance between the virtual plan and the postoperative positioning of bony segments.

## 4. Discussion

The results of the present study indicate that the integration of digital planning in bimaxillary orthognathic surgery is associated with improved three-dimensional accuracy in the repositioning of bony segments and a significant reduction in both preoperative and intraoperative time. Linear deviations between planned and postoperative positions were lower in the digital planning group (1.28–1.49 mm) compared with the conventional method (1.63–1.95 mm), with values remaining below the clinically accepted threshold of 2 mm in both cases [21,22]. This suggests that both methods allow predictable functional outcomes; however, the digital workflow appears to reproduce the virtual plan with greater fidelity. In addition, mean planning time was reduced by approximately 35% and operative time by approximately 34%, highlighting the practical advantages of computer-assisted planning [23,24,25].

Total RMS values remained below 2 mm for both methods, consistent with most recent studies that consider deviations below this threshold clinically negligible [17,26,27]. Previous research has shown that both the maxilla and mandible can be virtually repositioned with mean accuracy below 2 mm across all three spatial axes, confirming the reproducibility of digital planning [18,28,29]. The results of the present study fall within this range, with an average difference of approximately 0.4 mm in favor of the digital workflow, similar to findings reported by Strujak et al. [13], who observed a reduction in mean deviation from approximately 1.9 mm in conventional planning to approximately 1.5 mm in virtual planning. Other recent studies report comparable values, with Kwon et al. [30] describing mean deviations of approximately 0.95 mm and Ritto et al. [31] reporting values between 0.9 and 1.4 mm, confirming that the present results align with current international standards of accuracy.

Analysis based on anatomical landmarks revealed slightly lower deviations at A-point compared with Pogonion, suggesting better predictability of maxillary repositioning. Maxillary stability is influenced by its relationship to the cranial base and the use of intermediate splints, whereas mandibular positioning is more sensitive to variations in condylar positioning and autorotation phenomena. Similar findings were reported by Locmele et al. [32], who observed mean deviations of 0.71 mm for the maxilla and 0.91 mm for the mandible in 50 cases of bimaxillary surgery, supporting the reproducibility of digital planning even in complex procedures.

Another notable observation was the reduced variability of results in the digital group, indicating greater consistency in surgical plan transfer. Nilsson et al. [14] also reported lower variability in three-dimensional deviations when the entire workflow is digitalized. The elimination of analog steps—such as impression taking, articulator mounting, and manual splint adjustment—reduces the risk of cumulative errors. In digital workflows, these steps are replaced by virtual models and 3D-printed guides, allowing more accurate reproduction of the surgical plan. The integration of CBCT data with intraoral scans in dedicated software, such as IPS CaseDesigner, contributes to improved intraoperative precision and reduced risk of positioning errors [33].

A key advantage highlighted by the present results is the reduction in workflow duration. Mean planning time was approximately 57 min shorter in the digital workflow (106 ± 14 min vs. 164 ± 17 min), corresponding to a reduction of about 35%. Similar findings were reported by Alkaabi et al. [34], who demonstrated that virtual planning significantly reduces preparation time compared with conventional methods. Other studies have reported reductions of approximately 30% in planning time through the use of virtual simulations and real-time adjustment of bony segments [35]. After overcoming the initial learning curve, the digital workflow becomes faster and more intuitive, eliminating time-consuming laboratory steps.

The reduction in operative time represents another clinically relevant finding. In the present study, the mean operative duration was reduced by more than 90 min in the digital group. This difference should be interpreted cautiously, as the duration of individual intraoperative steps was not recorded. A more plausible explanation is a cumulative effect of several factors: more accurate transfer of the surgical plan, reduced need for intraoperative occlusal verification and segment repositioning, fewer adjustments of intermediate and final splints, and a more streamlined surgical workflow once the maxillomandibular relationship has been predefined virtually [36,37,38,39]. At the same time, because the digital workflow was implemented later, part of this difference may also reflect temporal optimization of the surgical protocol and increased team experience.

Beyond quantitative improvements, digital planning offers additional advantages related to three-dimensional visualization of craniofacial structures and simulation of osteotomies [40,41,42]. These tools allow anticipation of potential challenges, such as bony interferences or occlusal discrepancies, which may be difficult to assess using conventional methods [43,44]. Furthermore, the digital environment facilitates interdisciplinary collaboration between surgeon, orthodontist, and technician, enabling refinement of the treatment plan prior to surgery [45].

A novel aspect of this study is the analysis of the relationship between time efficiency and three-dimensional accuracy. The identified correlations indicate that reducing planning and operative time is not associated with decreased precision; on the contrary, it is associated with smaller deviations from the virtual plan. This finding suggests that the digital workflow not only streamlines the process but also enhances the predictability of surgical outcomes.

### 4.1. Limitations of the Study

The results should be interpreted in light of certain methodological limitations. The study included only bimaxillary orthognathic surgery cases (Le Fort I combined with BSSO), which are characterized by higher complexity and more challenging three-dimensional control of bony segments. Therefore, the findings reflect the performance of the two methods in a demanding clinical context and cannot be directly extrapolated to monomaxillary procedures or less severe cases.

Although baseline characteristics were comparable between groups, the non-randomized design does not allow complete exclusion of factors that may influence repositioning accuracy, such as individual anatomical variability, degree of asymmetry, or surgical complexity. In clinical practice, these variables may affect both planning and surgical execution, regardless of the method used.

Because group allocation followed a chronological transition in workflow rather than randomization, temporal confounding cannot be excluded. Improvements in team experience, workflow optimization, or perioperative management over time may have influenced operative efficiency and, to a lesser extent, accuracy outcomes.

Three-dimensional accuracy was assessed by CBCT superimposition. Although widely used, this method may be influenced by image quality, landmark identification, and minor variations in patient positioning during acquisition. In conventional planning, digitization of analog steps involves conversion of physical models into virtual format, which may introduce additional variability related to model alignment. Moreover, the condylar position and rotational deviations (yaw, pitch, roll) were not evaluated, although they may influence mandibular accuracy in bimaxillary surgery.

The use of A-point and Pogonion provides a simplified representation of three-dimensional accuracy. Rotational discrepancies, transverse asymmetry, occlusal plane changes, and condylar positioning were not directly evaluated. Although the inclusion of RMS offers a global assessment of deviation, a more comprehensive analysis would require additional anatomical landmarks and multi-planar evaluation.

Postoperative evaluation at 6–8 weeks was chosen to minimize the influence of edema and capture early stabilization of bony segments. However, this timeframe does not allow assessment of long-term stability, including changes due to bone remodeling or neuromuscular adaptation.

Axis-specific deviations (anteroposterior, vertical, and transverse components) were not analyzed. Therefore, the results reflect overall 3D discrepancies without directional decomposition, which limits comparability with studies using axis-based reporting.

Operator experience with digital planning software may influence initial workflow duration, as a learning curve is required. As familiarity increases, time differences may become even more pronounced.

Also, the direction of error (overcorrection versus undercorrection) was not analyzed, which limits the clinical interpretation of the observed deviations.

Patient allocation was not randomized; however, baseline comparability was confirmed for age, sex, and skeletal class. Rotational components and condylar position were not evaluated.

Despite these limitations, the results show consistent trends regarding the relationship between planning method, three-dimensional accuracy, and time efficiency, suggesting that digital integration may enhance surgical predictability.

### 4.2. Future Directions

The findings suggest that digital planning may improve the predictability of bimaxillary orthognathic surgery; however, further studies are needed to evaluate long-term stability. Extended follow-up could reveal adaptive changes in bony segment positioning or relapse tendencies, which are essential for assessing final outcomes.

Analysis of soft tissue changes would also be valuable, as aesthetic outcomes depend not only on skeletal repositioning but also on postoperative adaptation of facial structures. Correlating three-dimensional skeletal parameters with clinical facial changes could provide a more comprehensive evaluation of digital planning benefits.

Incorporating patient-reported outcomes could offer additional insight into perceived results, postoperative comfort, and treatment acceptance. Furthermore, cost-effectiveness analyses are needed to determine whether reduced operative time and increased predictability offset the initial investment in digital technologies.

Future advancements may include integration of surgical navigation systems, patient-specific guides, and intraoperative visualization tools to further improve accuracy. As these technologies become more accessible, digital workflows are likely to expand, although further validation in larger cohorts is required.

### 4.3. Clinical Relevance

Fully digital planning was associated with improved accuracy and reduced operative time in bimaxillary orthognathic surgery. These findings support the use of digital workflows to enhance procedural predictability. However, long-term stability and patient-centered outcomes require further investigation.

## 5. Conclusions

Fully digital planning in bimaxillary orthognathic surgery was associated with reduced three-dimensional deviations between the virtual plan and the postoperative position of bony segments compared with the conventional method. The observed submillimetric differences suggest improved concordance between planning and surgical outcome in the digital workflow.

At the same time, digital planning was associated with a significant reduction in both preoperative and operative time, indicating improved workflow efficiency. The identified correlations suggest that shortening planning and operative durations does not compromise the accuracy of surgical plan transfer.

The findings of this study are specific to bimaxillary orthognathic surgery and should be interpreted within this context. Further studies are needed to evaluate long-term stability and to extend the applicability of these observations to other types of orthognathic procedures.

## Figures and Tables

**Table 1:** Characteristics of the study sample.

Variable	Total Sample (*n* = 100)
Age (years)	
mean ± SD	25.02 ± 5.03
Gender, *n* (%)	
• female	54 (54.0%)
• male	46 (46.0%)
Skeletal class, *n* (%)	
• Class II	58 (58.0%)
• Class III	42 (42.0%)
Study group, *n* (%)	
• conventional	50 (50.0%)
• digital	50 (50.0%)

**Table 2:** Comparison between conventional and digital groups.

Variable	Conventional (*n* = 50)	Digital (*n* = 50)	*p*-Value
Age (years), mean ± SD	24.68 ± 5.66	25.36 ± 4.36	0.502
Gender, *n* (%)			0.688
• female	28 (56.0%)	26 (52.0%)	
• male	22 (44.0%)	24 (48.0%)	
Skeletal class, *n* (%)			0.685
• Class II	28 (56.0%)	30 (60.0%)	
• Class III	22 (44.0%)	20 (40.0%)	

**Table 3:** Comparison of 3D accuracy between conventional and digital planning.

Variable	Conventional (*n* = 50)	Digital (*n* = 50)	*p*-Value
RMS total (mm), mean ± SD	1.80 ± 0.54	1.39 ± 0.39	<0.001
A-point deviation (mm), mean ± SD	1.63 ± 0.36	1.28 ± 0.28	<0.001
Pogonion deviation (mm), mean ± SD	1.95 ± 0.44	1.49 ± 0.42	<0.001

**Table 4:** Comparison of time efficiency between conventional and digital planning.

Variable	Conventional (*n* = 50)	Digital (*n* = 50)	*p*-Value
Planning time (min), mean ± SD	163.92 ± 17.05	106.36 ± 13.89	<0.001
Operative time (min), mean ± SD	277.40 ± 28.29	184.24 ± 21.16	<0.001

**Table 5:** Multiple regression analysis for RMS deviation.

Predictor	B	β	*p*-Value
Group (digital vs. conventional)	−0.867	−0.847	<0.001
Age	0.019	0.188	0.039
Skeletal class	0.073	0.070	0.435
Model statistics: R^2^ = 0.250; Adjusted R^2^ = 0.218; F(4,95) = 7.898; *p* < 0.001			

## Data Availability

The original contributions presented in this study are included in the article. Further inquiries can be directed to the corresponding author.

## Abbreviations

The following abbreviations are used in this manuscript:

BSSO	Bilateral Sagittal Split Osteotomy
CBCT	Cone-Beam Computed Tomography
CI	Confidence Interval
CT	Computed Tomography
ICC	Intraclass Correlation Coefficient
IPS	Intelligent Planning System (KLS Martin CaseDesigner)
RMS	Root Mean Square (error)
SD	Standard Deviation
